# Complete Vision Loss after Laparoscopic Hysterectomy

**DOI:** 10.1155/2021/6643703

**Published:** 2021-02-28

**Authors:** Steven Radtke, Elizabeth Florence, Alexander Clavijo, Linh Do, Isabel Lopez

**Affiliations:** Department of Obstetrics and Gynecology, Paul L. Foster School of Medicine, Texas Tech University Health Sciences Center, 4801 Alberta Avenue, El Paso, Texas, USA 79905

## Abstract

Postoperative vision loss (POVL) is a rare but devastating complication that has only recently been reported following laparoscopic surgery. We present the case of a 34-year-old gravida 6 para 4 female who experienced POVL following an uncomplicated laparoscopic hysterectomy. Operating time was 174 minutes, and EBL was 75 mL. After surgery, she complained of complete vision loss with no light perception. No cerebral hemorrhage or ischemia was detected on imaging. Funduscopic exam revealed no structural abnormalities. On postoperative day 7, she received an IV methylprednisolone taper. The following morning, she reported mild light perception. Later that night, she reported a partial return of visual acuity and was discharged home. At her 2-week postoperative visit, her vision had returned to baseline. POVL is an emergency and prompt evaluation should be initiated to optimize outcome.

## 1. Introduction

Perioperative vision loss (POVL) following nonocular surgery is a rare but devastating and unexpected complication with limited reports in the minimally invasive surgery literature. We present the case of a 34-year-old female who underwent an uneventful laparoscopic hysterectomy and suffered from complete blindness following the operation. We subsequently describe the most common etiologies and how they pertain to the presented case.

## 2. Case Presentation

A 34-year-old G6P4024 initially presented with symptomatic abnormal uterine bleeding and severe dysmenorrhea of three years duration. Based on her evaluation, which included a detailed history, pelvic exam, and ultrasound, endometriosis and adenomyosis were suspected. She had attempted medical management with multiple regimens including combined oral contraceptives, megestrol acetate, and tranexamic acid without resolution of symptoms. She had completed her family planning after four uncomplicated vaginal deliveries and two spontaneous abortions. Given that her quality of life was impaired to a significant degree, she opted for definitive surgical management and a laparoscopic hysterectomy was planned.

Her medical history was remarkable for anxiety, depression, and endometriosis. Previous surgical interventions included removal of a right breast lump, cholecystectomy with removal of a benign liver mass, and laser ablation of extensive pelvic endometriosis. She had no known drug allergies. She denied history of abnormal cervical cytology or sexually transmitted diseases. Her BMI was 39.1 kg/m^2^, and physical exam was remarkable for lower abdominal pain and mild tenderness to palpation. Exam and vitals were otherwise within normal limits. An endometrial biopsy was performed as part of her evaluation, and it revealed proliferative endometrium with no hyperplasia or malignancy. Preoperative laboratory values were unremarkable (Table 1).

She underwent a total laparoscopic hysterectomy, bilateral salpingectomy, and excision of deeply infiltrating endometriosis from the right pelvic sidewall. General anesthesia was induced with propofol and maintained with desflurane. She was in the dorsal lithotomy position on a gel pad with both arms tucked. The operation was uncomplicated. For laparoscopy, the abdomen was insufflated with carbon dioxide gas to a pressure of 15 mmHg. The total operating time was 172 minutes, out of which approximately 100 minutes were in the Trendelenburg position at an angle of 21 degrees. The estimated blood loss was 75 mL. She received approximately 1400 mL of crystalloid solution during the case.

After surgery, the patient was transferred to the PACU for recovery. She reported feeling somewhat groggy, as well as moderate right shoulder discomfort. Her vital signs remained within acceptable range. Upon arrival to the surgical floor, approximately 4 hours postoperative, she became increasingly distressed, complaining that she was unable to see. A stroke code was activated, and she was immediately evaluated by the on-call neurology team. At this time, she reported no perception to light. She described only being able to “see” a white wall. Her physical exam revealed normal strength and sensation in all extremities. Cranial nerves II-XII were intact. No focal neurologic deficits were found, and her pupils were equally reactive to light bilaterally. Optokinetic nystagmus was present. A stat CT of the head and neck was ordered and was negative for acute hemorrhage or ischemia.

**Figure 1:**
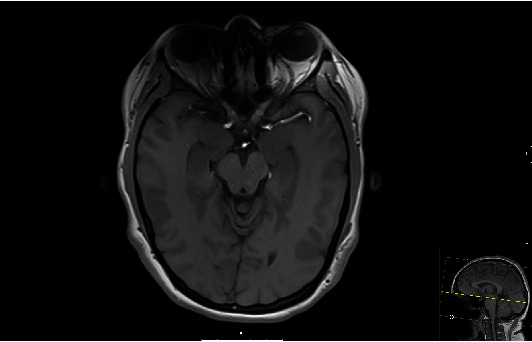
T1 sequence of axial view of brain. No cortical ischemia or hemorrhage was visualized. No evidence of compression or injury to optic nerve.

Her visual symptoms remained unchanged overnight. The next morning her laboratory values were within expected range (Table 1). She underwent a contrasted MRI of the brain which showed orbits within normal limits (Figure 1) and an incidental finding of a 5 × 3 mm eccentric nonenhancing intrasellar cystic lesion in the left pituitary gland as well as scatter foci of T2/FLAIR signal hyperintensities in the subcortical and deep white matter of bilateral frontal lobes read as nonspecific findings in patients with chronic migraines. The radiologist acknowledged that the evaluation of the orbits was somewhat limited secondary to orthodontic hardware.

The patient was evaluated by the ophthalmology service. Their examination revealed a normal range of motion in both eyes, and pupils were equally round and reactive to light. Tonometry demonstrated a normal intraocular pressure of 18 mmHg in the left eye and 13 mmHg in the right. The lenses were clear, maculae flat, and periphery was within normal limits bilaterally. Her acuity of vision remained classified as no light perception. Outpatient follow up with neuroophthalmology and an inpatient psychiatry consult were recommended. They suggested a diagnosis of intracranial vs. factitious disease.

The psychiatry service examined her on the third day after surgery. Based on their evaluation, they could not rule out an organic cause for the blindness and recommended further evaluation by neurology and ophthalmology. They noticed some symptoms of anxiety and started her on duloxetine 20 mg daily.

Over the following days, her vision remained unchanged. She continued to describe a white wall and was unable to detect motion or light. Otherwise, she met all appropriate postoperative milestones from a gynecologic perspective. Her vitals remained stable, and she was afebrile. Surgical pain was minimal, and laparoscopic port sites remained clean, dry, and intact. She was ambulatory with assistance and was voiding without difficulty. Laboratory values also remained stable. Given recent postoperative status, inflammation markers were not obtained. A bilateral carotid Doppler was ordered on postoperative day 5 to evaluate for a possible embolic source or stenosis, but this was negative. She was able to sign her name on a piece of paper and touch her index fingers together when prompted to do so. On postoperative day 7, given that her vision was not improving, a steroid taper trial was discussed. She was counseled on the potential risks and the fact that there was no clear evidence of a steroid responsive condition. After agreeing to proceed, she received IV methylprednisolone for 3 doses separated by 6 hours each, tapered from 60 mg, to 40 mg, then 20 mg. She reported feeling “eye heaviness” during this time, but no changes in vision.

The following morning (postoperative day 8), she reported that she was able to perceive certain changes in light. This was confirmed on exam, where she was able to tell if a flashlight was moving in-front of her eyes. At this point, her visual acuity had improved to light perception only. During the night, the patient reported her vision had spontaneously returned. Acuity was blurry, but she was able to identify objects and movement. She was discharged later that day with instructions to follow up in a week.

At her postoperative visit in clinic, she noted that her vision continued to improve after discharge and was now resolved to her baseline. Her uncorrected Snellen eye exam was 20/63 for her left eye and 20/50 for her right.

## 3. Discussion

POVL following nonocular surgery is a rare but devastating complication that can have significant physical and socioeconomic implications. Because of this, it should be considered a medical emergency, leading to a prompt multidisciplinary evaluation. The increasing number and complexity of cases performed via the minimally invasive approach may lead to a rise in complications related to long operating times in the Trendelenburg position [1]. Awareness of the physiologic changes and potential impact this can have may prompt taking preventive measures that mitigate some of the risks.

### 3.1. Corneal Abrasion

Corneal damage is one of the most common insults to the optic organs following nonocular surgery. The mechanism of injury is secondary to either direct mechanical trauma or exposure keratopathy (deficient taping of the eyelids) [2]. Incidence can vary depending on the diagnostic criteria utilized. It has been found to be as high as 44% when diagnosis is made via fluorescein staining; however, only 0.17% is symptomatic [2]. Presentation usually consists of unilateral tearing, miosis, photophobia, and foreign body sensation. Full vision loss is rare. This is not consistent with the described case, in which irritation symptoms were absent, and vision loss was bilateral and complete.

### 3.2. Cortical Visual Loss

Cortical visual loss is another relatively common etiology of POVL. In a nationwide retrospective study spanning a 10-year period, incidence was found to be 0.38 per 10,000 discharges [3]. Cortical blindness (CB) usually occurs as a result of cerebral infarction from embolism, most often of the posterior cerebral artery. This condition is most commonly seen after surgeries with a high risk of cerebral embolism such as cardiac and spinal surgery [3]. Carotid stenosis or dislodgement of carotid plaque can also serve as the embolic source. A Doppler ultrasound of the carotid arteries was negative in this case. CB can also result from intracranial hemorrhage, focal ischemia secondary to hypoxia, or low blood volume due to hemorrhage. Pupillary reflexes are usually preserved, as they were in the described case. However, a CT scan and contrasted MRI were thoroughly reviewed, and there were no signs of hemorrhage or ischemia.

### 3.3. Ischemic Optic Neuropathy (ION)

ION occurs when intraoperative factors either decrease perfusion pressure to the optic nerve, or when there is an increase in intraocular pressure (IOP), which in turn augments resistance to perfusion [4]. The typical presentation for perioperative ION is sudden painless visual loss after surgery [5]. It is often unilateral. Prolonged operating time in steep Trendelenburg has been considered a risk factor for increased IOP which can lead to ION [6]. Additional risk factors include hypotension, anemia, edema, venous congestion, and excessive fluid resuscitation [7]. Despite these being accepted risk factors, there is no clear evidence that counteracting them can prevent the condition.

Although this was part of our differential diagnosis, there were several elements that decreased the likelihood of this possibility. Having bilateral ION is not a common presentation. Additionally, blood loss during surgery was minimal, and the Trendelenburg position was held for less than two hours. Fundoscopic exam by ophthalmology did not reveal edema or swelling of the optic disc, which can sometimes be witnessed in cases of anterior ION. However, in posterior ION, there are usually no changes until weeks after the insult [4]. The patient did have adequate pupillary reflexes, which are usually not evidenced in ION.

### 3.4. Central Retinal Artery Occlusion (CRAO)

The pathogenesis of POVL due to CRAO involves retinal damage following decreased perfusion from the central retinal artery due to external compression of the eye. This is usually caused by improper intraoperative head positioning. Vision loss is unilateral. Other findings on exam include periorbital and eyelid edema, chemosis, proptosis, ptosis, attenuated retinal vessels, and loss of eye movements [8]. Prone positioning increases the risk of external eye compression. This presentation is more common in operations that require this position, which is not routinely used in gynecologic surgery. There were no signs on physical exam or imaging suggesting eye inflammation or retinal blanching in this case. Additionally, the bilateral presentation in a procedure with no risk factors for intraoperative eye compression made the diagnosis unlikely.

### 3.5. Nonorganic Vision Loss (NOVL)

Postoperative NOVL is a diagnosis of exclusion that is made once all anatomic sources and pathways of visual impairment have been ruled out. This condition is more common in younger patients and diagnosed more frequently in females. It encompasses a wide array of conditions, from malingering to psychogenic (conversion disorder) [9]. There are several clinical tests that can be used to differentiate the patient who is falsely claiming visual loss for secondary gains vs. the patient who is not in control of their symptoms. The name-signing test is a clear example of this. The patient is asked to sign their name on a piece of paper. A malingering patient will usually claim they are unable to do so, or produce ineligible scribbles, whereas someone with organic or psychogenic nonorganic blindness will do so with minor difficulties at most [9]. A similar test consists of asking patients to spread their arms out and then join their index fingertips. A patient who is intentionally faking visual loss will act as if they are unable to complete the task and exaggerate their inability to do so. A nonmalingering blind patient will still be able to complete the task effectively secondary to proprioception. Between 45% and 78% of patients with NOVL experience complete resolution of symptoms, although time to recovery can range from a few days to months [10].

During the evaluation of the presented patient, she was able to sign her name and touch her fingertips together without difficulty, which made the diagnosis of malingering less likely. A psychogenic cause for vision loss was considered.

## 4. Conclusion

Treating POVL as an emergency is of paramount importance given the severe implications the diagnosis can carry. A prompt multidisciplinary approach allows for rapid identification of potential etiologies and the development of a targeted treatment strategy.

## Figures and Tables

**Table 1:** Laboratory values.

Laboratory test	Preoperative	POD#1	POD#2	POD#3
WBC (K/*μ*L)	6.41	9.68	10.74	6.70
Hemoglobin (g/dL)	11.4	11.3	9.7	9.8
Hematocrit (%)	35.9	34.5	30.7	31.5
Platelets (K/*μ*L)	312	311	257	234
Na (mmol/L)		135	138	
K (mmol/L)		4.4	3.5	
Cr (mg/dL)		0.5	0.6	

POD: postoperative day; WBC: white blood cells.

## Data Availability

The data presented in this study pertained to only one patient, and relevant de-identified data is presented in the table included in the manuscript.
